# Structural Aesthetic Treatment With the Hyaluronic Acid Filler VYC‐25L: Global Expert Considerations for Safe and Effective Long‐Term Outcomes

**DOI:** 10.1111/jocd.16555

**Published:** 2024-11-26

**Authors:** Dario Bertossi, Radina Denkova, Anna Jen Shi Hoo, David Loh, Marshall Murdoch, Isaac Shturman Sirota, Fernando Urdiales‐Gálvez, Marcel Vinícius de Aguiar Menezes, Carola de la Guardia

**Affiliations:** ^1^ Department of Maxillo Facial Surgery, Head & Neck Department Università degli Studi di Verona Verona Italy; ^2^ Dr. Denkova Dermatology Sofia Bulgaria; ^3^ Anna Hoo Clinic Kuala Lumpur Malaysia; ^4^ David Loh Surgery Singapore Singapore; ^5^ Knysna Advanced Health Medical Centre Knysna South Africa; ^6^ Division of Plastic Surgery University of Cape Town Cape Town South Africa; ^7^ Plastica Shturman, Hospital Angeles Lomas Huixquilucan de Degollado Mexico State Mexico; ^8^ Instituto Médico Miramar Málaga Spain; ^9^ Cosmiatry for Doctor Aracaju Sergipe Brazil; ^10^ Global Aesthetics Medical Affairs, Allergan Aesthetics, an AbbVie Company Madrid Spain

**Keywords:** duration, facial contouring, hyaluronic acid filler, jawline definition, structural product, volume restoration, VYC‐25L

## Abstract

**Background:**

VYC‐25L is a robust, structural hyaluronic acid (HA) filler designed for facial volumizing, lifting, and contouring. It was first approved in 2019.

**Methods:**

A group of doctors with various specialties, who have used VYC‐25L extensively since it first became available in their countries (3–5 years), share clinical experience and guidance on optimal use.

**Results:**

VYC‐25L has a unique rheological and physicochemical profile that provides elevated lift capacity and enhanced projection, significant moldability immediately after injection, high levels of tissue integration, reversibility with hyaluronidase, and a long duration of clinical effects—typically lasting at least 24 months. The properties of VYC‐25L have created new possibilities for nonsurgical facial medical aesthetics. However, as with any novel product, it is important that injectors recognize how best to use it for the benefit of patients. When first utilizing VYC‐25L, it is advisable to start with the chin and jawline to gain familiarity with the gel characteristics before moving into other facial areas, and to consider splitting treatment over two or more sessions. Attention must also be given to injection volume, with less product typically required with VYC‐25L compared to other fillers with similar indications. Key principles of good practice should be followed, including appropriate patient selection and pretreatment education, suitable choice of injection device and plane, aseptic technique, slow and careful administration method, and sufficient posttreatment follow‐up.

**Conclusions:**

By adhering to these principles, VYC‐25L can produce natural‐looking and highly durable outcomes without substantial safety concerns.

## 1 Introduction

Nonsurgical facial contouring has evolved significantly as new products have come to the market. VYC‐25L (Juvéderm VOLUX, Allergan Aesthetics, an AbbVie Company, Madison, NJ) is an innovative structural hyaluronic acid (HA) filler first launched in 2019, based on the Vycross technology [1]. It has a unique rheological and physicochemical profile—including high levels of cohesivity and a high elastic modulus (G′)—which yields enhanced lifting capacity and projection, improved resistance to deformation, and a long duration of effect [2, 3]. These characteristics make VYC‐25L an excellent choice for restoring and creating volume, as well as for structural improvement.

In a prospective, randomized controlled study of 119 individuals with chin retrusion, VYC‐25L treatment was associated with significant improvements in facial angle, patient‐ and investigator‐assessed aesthetic enhancement of the chin and jawline, and high levels of patient satisfaction [2]. These effects were typically durable out to the time of re‐treatment (18 months) and no serious adverse events were recorded [2, 4].

The product was first launched in 2019 in Europe, with further approvals then following across the world. Subsequent real‐world studies confirmed the safety and effectiveness of VYC‐25L in routine practice for treatment of the chin and jawline, as well as the nose [5, 6, 7, 8, 9]. Furthermore, a prospective ultrasound study demonstrated that 2 days after administration, there was partial biointegration of HA into the tissue of the lower face, and total integration was achieved within 30 days [10].

The properties of VYC‐25L are opening up new avenues of possibility for facial aesthetic improvement with HA fillers. However, this also creates novel challenges for injectors in understanding how best to employ the product for the benefit of patients. The objective of the current paper is to share clinical experience and advice on optimal use of VYC‐25L—as well as key considerations for safe outcomes—from a global group of doctors with various specialties who have used the product extensively over the past 3–5 years.

## 2 Methods

In December 2023, all authors (except for the last author, who is an employee of AbbVie) independently completed a written questionnaire on their personal experiences with VYC‐25L. This was divided into five separate sections covering: levels of usage; initial experience of the product (first 6 months), recent experience of the product (past 6 months), injector preferences, and patient management. Later that month, each one was separately interviewed via video call to clarify and elaborate on the written responses. The present paper collates the joint experience of the entire group from countries around the world.

At the time of completing the questionnaire, all injectors had between 3 and 5 years of experience with VYC‐25L (depending on the date of product approval in their country). We estimate that we have treated more than 6000 patients in total using this product.

### 2.1 Key Features of VYC‐25L

For each of the injectors, our top three reasons for preferring VYC‐25L over other structural products are listed in Table 1. The most commonly cited reasons relate to the rheological and physicochemical properties of VYC‐25L (*n* = 6/8); long duration of effects (*n* = 6/8); ease and smoothness of injection (*n* = 3/8); and reversibility with hyaluronidase if needed (*n* = 2/8). Other factors listed by one author each include general familiarity with the Vycross range, product moldability, integration capacity, and structural support.

**TABLE 1:** Top three reasons for choosing VYC‐25L over other structural products.

Author^a^	Top three reasons for using VYC‐25L		
DB	Smoothness of injection	Duration of effect	Lack of palpability over time
RD	Implant‐like rheological and physicochemical properties	Structural support	Projection capacity
AJSH	Implant‐like rheological and physicochemical properties	Ease of injection	Duration of effect
DL	Implant‐like rheological and physicochemical properties	Reversibility (hyaluronidase)	Familiarity with Vycross range
MM	Reversibility (hyaluronidase)	Rheological and physicochemical properties	Duration of effect
ISS	Rheological and physicochemical properties	Low extrusion force	Duration of effect
FUG	Duration of effect	Myomodulation capability	Product integration
MVdAM	Strength (firmness) of the product	Moldability	Duration of effect

^a^ Listed in alphabetical order by surname.

VYC‐25L is formulated to an HA concentration of 25 mg/mL with 0.3% w/w lidocaine [11], and has the rheological and physicochemical properties required of a robust structural product with elevated lifting capacity (Table 2). In particular, it has a high G´ (665 Pa at 5 Hz) [3], and can therefore deliver significant volumization and increased projection. Furthermore, the high cohesivity of VYC‐25L (93 gmf) [3] means that it does not break up once injected. This allows the administered product to resist compression forces and maintain its original shape once in situ. Despite its firmness, the gel is smooth and easy to inject, and does not require great extrusion force (16.5 N at 50 mm/min with the supplied syringe and 27G 1/2″ needle [data on file, Allergan Aesthetics]), which helps to maintain precise control of delivery—in terms of speed, volume, and plane of injection. Thus, treatment with VYC‐25L can provide contour and targeted definition. It is moldable immediately postinjection but almost implant‐like once administration is complete. The product integrates fully into the host tissue within a few weeks [10], and so yields natural‐looking results [12].

**TABLE 2:** Key rheological properties of Vycross products [3].

Product	HA (mg/mL)	G′_5Hz_ (Pa)	Cohesivity/Fn (gmf)	Maximum water uptake, %
VYC‐25L	25	665	93	253
VYC‐20L	20	398	40	227
VYC‐17.5L	17.5	340	30	184
VYC‐15L	15	271	19	133
VYC‐12L	12	166	12	<100

Abbreviation: HA, hyaluronic acid.

Overall, these features contribute to a product with a unique profile that cannot be found in any other filler currently available worldwide.

### 2.2 Clinical Experience and Technical Considerations

VYC‐25L can be used in different ways to achieve different patient goals—whether relating to rejuvenation, beautification, facial masculinization/feminization, or the correction of structural deficits like microgenia [13]. This facilitates individualization of outcomes. When we first started using the product, most of the current author group injected it primarily into the chin and jawline. This aligned with the data available at that time from a prospective multicenter trial [2, 4].

The areas that we now treat most often with VYC‐25L are shown in Table 3. Overall, the chin and jawline remain the most frequently injected, but several of us commonly use it in the temple, cheek, and nose. However, it is important to note that the risk of vascular compromise is particularly high in the nose, and VYC‐25L should be used in this area only by expert injectors for specific corrections where it may be advantageous over alternative HA fillers. Other areas treated with VYC‐25L by at least one author in smaller numbers of patients include the lateral orbital rim and marionette lines. Treatment of the latter was an optional element of the US pivotal trial [14]. Nonetheless, for practitioners who are new to the product, we recommend starting with the chin and jawline to gain familiarity with the gel characteristics before expanding into other facial areas.

**TABLE 3:** Facial areas treated most often with VYC‐25L.

Author^a^	Facial areas^b^		
DB	Chin (90%)	Jawline (90%)	Nose (20%–30%)
RD	Chin (80%)	Jawline (80%)	—
AJSH	Chin (90%)	Jawline (80%)	Cheek/Temple (10% each)
DL	Temple (70%–80%)	Cheek (70%–80%)	Chin (30%)
MM	Chin (80%–90%)	Jawline (30%)	Nose (10%)
ISS	Chin (100%)	Jawline (100%)	Nose (30%)
FUG	Temple (80%)	Cheek (80%)	Chin (70%)
MVdAM	Chin (100%)	Jawline (100%)	Nose (5%)

^a^ Listed in alphabetical order by surname.

^b^ Percentages indicate the estimated proportion of all VYC‐25L‐treated patients injected in that area.

Consideration must be given to injection volume. In our experience, VYC‐25L provides better contouring and greater lift than other available HA fillers—and these results can be achieved using less product with VYC‐25L than VYC‐20L (at least in the first treatment session). This may lead to cost reductions for patients and could have important technical implications for injectors. Practitioners transitioning from VYC‐20L who use equivalent volumes to address the same defect risk causing overcorrection, discomfort, and tenderness to the touch. Indeed, the firmness of VYC‐25L and the tightness of treatment areas like the chin mean that lower initial volumes are generally advisable, and a degree of caution is needed when first using VYC‐25L.

Regarding the technical specifications, we employ various different approaches in our personal practices. However, seven of the eight injectors in the current author group use the MD Codes developed by Mauricio de Maio at least some of the time [15, 16]. The eighth injector (ISS) does not use this method and favors a column‐based injection technique.

The MD Codes system provides a systematic method for assessing and treating the face with JUVÉDERM fillers, including guidance on injection locations, layers, devices, delivery techniques, product selection/volume, and danger zones. A recent expert consensus from Spain and Portugal proposed a customized 7‐point protocol for women and a 9‐point pattern for men treated with VYC‐25L [13]. These were adapted from the work of de Maio [17], based on MD Codes for the chin, jawline, and cheek. Similarly, an Italian group has developed grid‐based approaches for treating the chin, jawline, and nose [5, 6].

Thorough consideration should be given to the choice of injection device and administration plane when using VYC‐25L, and these decisions should always be tailored to the specific treatment area. The product is supplied with a 27‐G needle, but either a needle or a cannula can be used in practice—and each offers particular advantages and disadvantages. For example, the sharpness of a needle may make it easier to direct into the desired plane of treatment [18], whereas the bluntness of a cannula might decrease the risk of penetrating a blood vessel [19]. Importantly, VYC‐25L administration has been shown not to require a large extrusion force with the supplied syringe and needle (data on file, Allergan Aesthetics), and hence narrow lumen diameters can be used (25–27G cannula; 27–30G needle). This allows smaller product volumes to be delivered and helps to improve the precision of placement, which may be particularly important for reducing the risk of nodule formation when injecting less deep.

Regarding the injection plane, VYC‐25L can be administered into the subcutaneous or supra‐periostal layers [11]. However, it is essential that any practitioner wanting to use VYC‐25L first develops a thorough personal understanding of facial anatomy. The choice of injection depth is closely linked with safety concerns relating to the likely positions of key blood vessels and nerve structures. The plane of deposition can also have implications for effectiveness outcomes. When injected deep (supra‐periosteal), the implant‐like properties of VYC‐25L replicate natural bony structures and can provide significant and durable volume enhancement without causing surface irregularity. When used subcutaneously, there is a greater risk of irregularity and visibility, and careful postinjection massage may be needed to ensure that the product is well distributed; VYC‐25L is moldable immediately after administration and can therefore be evenly distributed. With subcutaneous injections, it might also be worthwhile to under‐correct slightly to allow for small increases in volume due to water uptake over the following weeks.

When using larger amounts of product, practitioners should consider splitting their treatment plan over two (or even three) separate sessions. Such a phased approach can help to build a natural‐looking outcome and mitigate postinjection concerns, such as firmness, swelling, or tenderness.

An important additional possibility with products like VYC‐25L is derived from the high levels of structural support they provide—potentially allowing targeted increases or decreases in muscle action through precise positioning of the product [13, 20]. For example, in patients with a deep labiomental sulcus and a particularly strong mentalis muscle, treatment with VYC‐25L (chin point C1 in the MD Codes [15]) can be used both to fill the depression and to mechanically block overactive muscle action.

Irrespective of the aims of treatment, practitioners should always monitor outcomes over time. This can be done using high‐quality, standardized photography, ideally taken in frontal, oblique, and lateral views (right and left) without make‐up or jewelry and using a consistent background [13, 21]. There are also 3D imaging systems that can provide objective assessments of results.

### 2.3 Duration of Effect

**FIGURE 1:**
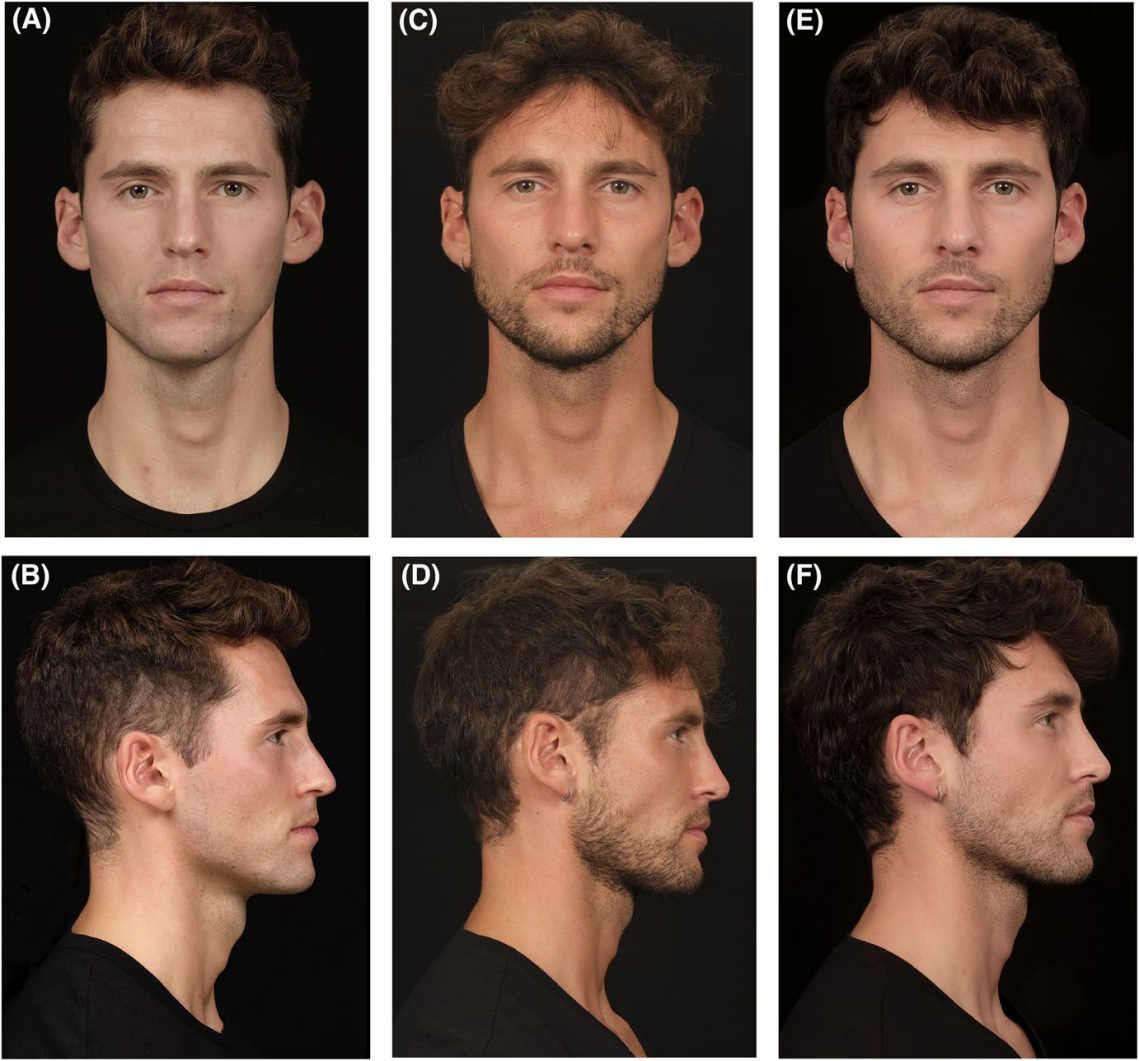
Durable improvements with VYC‐25L treatment. A 32‐year‐old male shown before (A, B), 1 month after (C, D), and 2 years after treatment with VYC‐25L (E, F). The patient was injected bilaterally with 1 mL in Jw1–3, 1 mL in Jw4–5, 0.3 mL in C1, and 0.3 mL in C5. He also received VYC‐20 in the nose. The long‐term impact of VYC‐25L treatment is particularly visible in the improved width and definition of the patient's jawline at 2 years postinjection. Anatomical locations are defined according to the MD Codes [15]. Images are courtesy of Dario Bertossi. C, chin; Jw, jaw.

**FIGURE 2:**
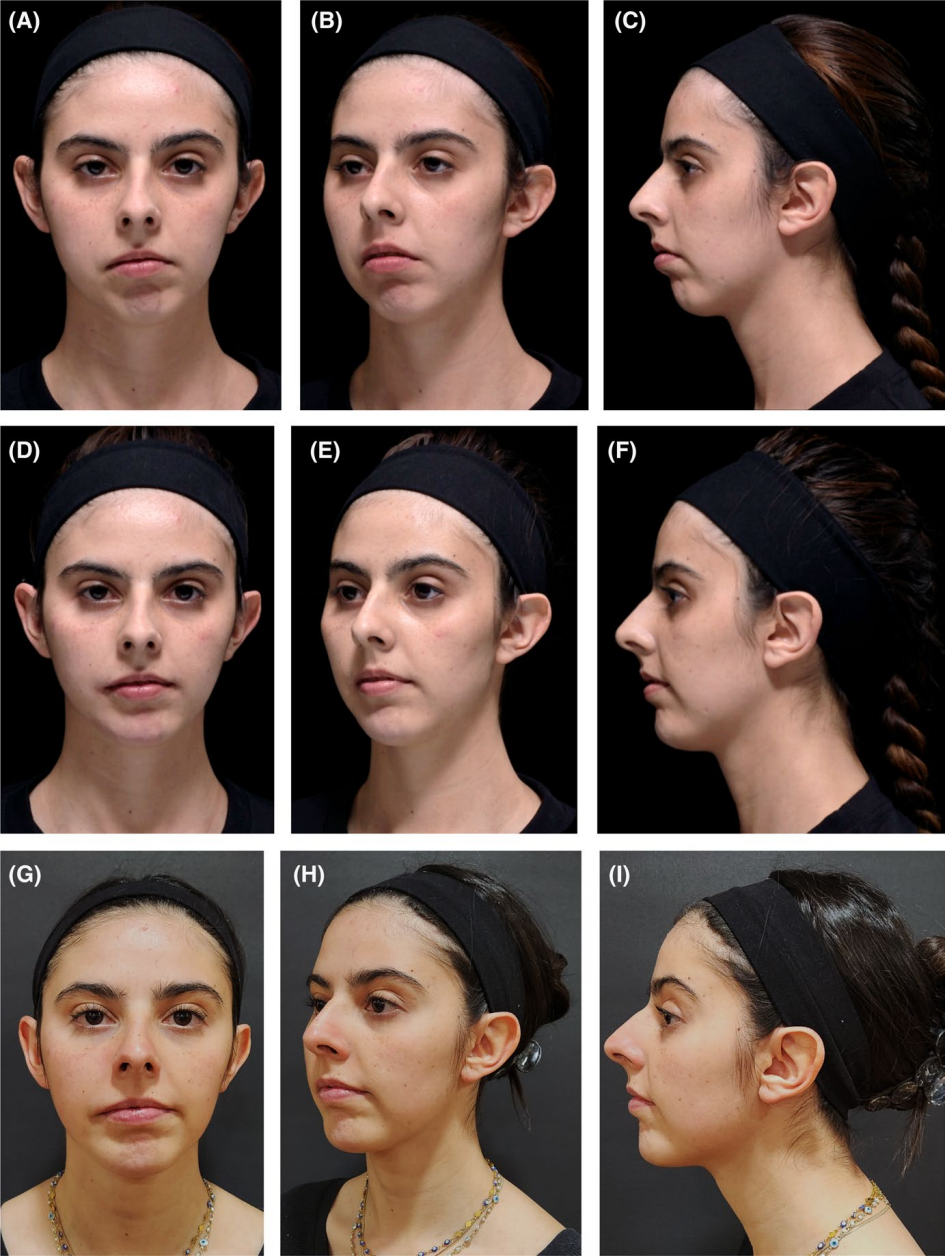
Durable improvements with VYC‐25L treatment. A 24‐year‐old female shown before (A–C), immediately after (D–F), and 3.2 years after treatment with VYC‐25L (G–I). The patient presented with Grade I left hemifacial microsomia. She was injected with 1 mL across points Jw1, 3, 4, and 5 on the right side; 0.7 mL across points Jw1, 3, 4, and 5 on the left side; 1 mL across points C3, 5, and 6 on the right side; 0.6 mL across points C3, 5, and 6 on the left side; and 0.7 mL in points C1, 2, and 4. She also received 2 mL of VYC‐20 across Ck1–3 and Lp6. The long‐term impact of VYC‐25L treatment is particularly visible in the increased projection and decreased leftward deviation of the patient's chin at 3.2 years postinjection. Anatomical locations are defined according to the MD Codes [15]. Images are courtesy of Isaac Shturman Sirota. C, chin; Ck, cheek; Jw, jaw; Lp, lip.

**FIGURE 3:**
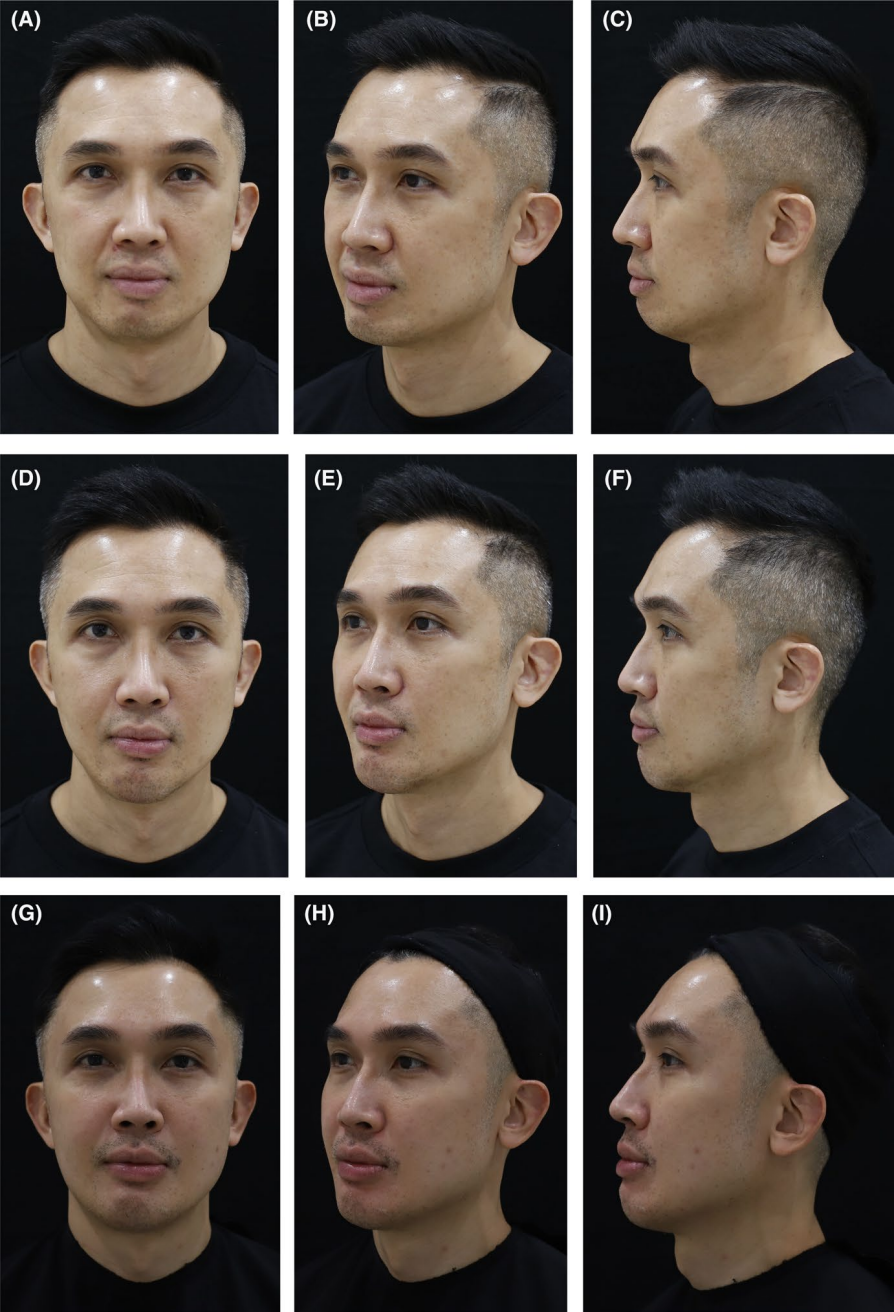
Durable improvements with VYC‐25L treatment. A 46‐year‐old male shown before (A–C), 1 month after (D–F), and 2 years after treatment with VYC‐25L (G–I). The patient was injected with 0.7 mL bilaterally in C1, 0.3 mL bilaterally in C2, 0.4 mL in C4, 0.3 mL bilaterally in C5, 0.5 mL bilaterally in Jw1, 0.5 mL bilaterally in Jw4, and 0.5 mL bilaterally in Jw5. He also received full‐face treatment using other products from the Vycross range but only VYC‐25L was used in the jawline and chin. The long‐term impact of VYC‐25L treatment is particularly visible in the increased projection of the patient's chin and the improved definition of his jawline at 2 years postinjection. Anatomical locations are defined according to the MD Codes [15]. Images are courtesy of Anna Hoo. C, chin; Jw, jaw.

**FIGURE 4:**
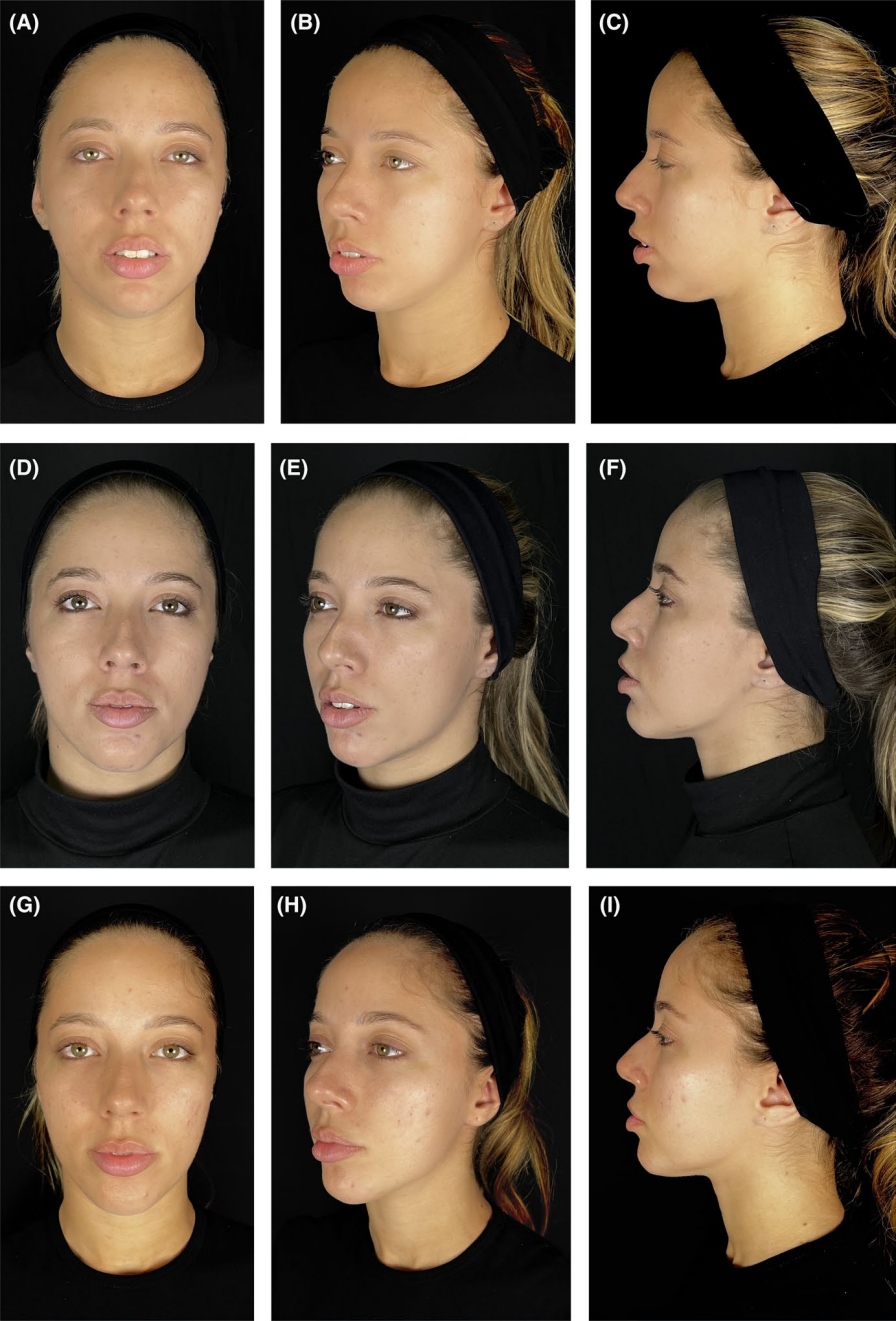
Durable improvements with VYC‐25L treatment. A 21‐year‐old female shown before (A–C), 2 months after (D–F), and 2 years after treatment with VYC‐25L (G–I). The patient was injected bilaterally with 0.3 mL in Jw1, 0.7 mL in Jw3, 0.5 mL in Jw4, 0.5 mL in Jw5, 0.5 mL in C1, 0.2 mL in C2, and 0.3 mL in C3. The long‐term impact of VYC‐25L treatment is particularly visible in the increased projection of the patient's chin and the improved definition of her jawline at 2 years postinjection. Anatomical locations are defined according to the MD Codes [15]. Images are courtesy of Marcel Vinícius de Aguiar Menezes. C, chin; Jw, jaw.

In a European prospective, randomized trial of VYC‐25L for contouring of the chin and jawline, patients were eligible for repeat treatment at 18 months, and outcome duration was not assessed beyond this timepoint [4]. However, in our experience, clinically meaningful effects typically persist for at least 24 months following VYC‐25L treatment (Figures 1, 2, 3, 4). Thus, the duration is at least as long as previously described with VYC‐20L [22, 23]. Moreover, when patients return for re‐injection, they often require smaller volumes of VYC‐25L to generate similar outcomes to their initial treatment.

In general, the duration of effect with VYC‐25L is longer in less mobile areas of the face, such as the nose, as compared to areas with more muscle activity and high compressive/shear forces, like the chin. Relaxation of the muscles in the chin using botulinum neurotoxin type A (BoNTA) may therefore increase the duration of filler effects in this area. However, we would only advocate BoNTA use if it is specifically indicated.

We have also noted a range of other factors that can impact on VYC‐25L duration. For example, clinical effects may be somewhat reduced in patients with elevated metabolism (such as from frequent high‐intensity exercise); those with excessive free‐radical generation (from smoking or photoaging); or individuals receiving energy‐based treatment for skin tightening near the filler‐injected area (radiofrequency, pulsed light, or high‐frequency ultrasound). Severe systemic infection can also detrimentally affect outcome duration. On the other hand, maintaining a healthy lifestyle—including good nutrition, adequate hydration, sufficient sleep, and low levels of stress—may help to maximize duration, as with all HA fillers. Age does not necessarily have a significant effect, although results may *appear* less durable in some older individuals if they have high levels of skin laxity.

### 2.4 Safe Outcomes With VYC‐25L

In skilled hands, VYC‐25L is not difficult to use and need not generate any more safety concerns than other HA fillers. Nonetheless, it is primarily a product for advanced injectors, and there are important considerations for safe outcomes.

Before treating their own patients with VYC‐25L, practitioners should: obtain adequate training in injection techniques, understand the product's physicochemical and rheological properties, and build expert levels of knowledge of facial anatomy, particularly in relation to the positioning of major blood vessels. For less experienced injectors, a step‐by‐step approach to professional development should be considered—based on starting by using VYC‐20L to treat the chin and jawline, before graduating to using VYC‐25L in these indications, and then considering other potential treatment areas with VYC‐25L.

As with all fillers, a thorough patient history (e.g., recent systemic disease, surgery, dental work, or vaccination) and examination should be completed before proceeding with treatment. Appropriate patient selection is also important. For example, some of the present author group advise choosing a product with lower G′, such as VYC‐20L, for individuals with particularly thin skin or a lack of fat mass in the treatment area; using VYC‐25L in such patients may carry an increased risk of injection‐site reactions like pain or swelling, and there could also be a greater chance of surface irregularities. Other authors note that with careful consideration of injection volume and depth, VYC‐25L can often still be used in these individuals.

As already described, when planning to use large volumes of VYC‐25L, practitioners should consider splitting this across multiple treatment sessions. Administration of excessive quantities can lead to over‐stretching of the tissue and compression of the microvasculature, causing pain and an increased risk of other complications. In addition, attention should always be given to the choice of injection device and plane with VYC‐25L, and this decision should be specific to the treatment area. From a safety perspective, the key consideration is avoiding major blood vessels—such as the facial artery in the jawline, the mental and labiomental arteries in the chin, and the nasal arteries within the nose.

As with any HA filler, and particularly owing to the long duration of VYC‐25L, aseptic technique is essential for avoiding late‐onset complications [21]. This should include complete removal of make‐up, thorough cleaning of the treatment area with an effective antiseptic (e.g., alcoholic chlorhexidine), use of gloves, and avoidance of touching the needle/cannula.

To minimize pain and reduce the risk of vascular trauma, it is essential to be gentle with the injection device following insertion, administer the product slowly using minimal force, and deposit small amounts in each area. Posttreatment pain can be an issue, for example when performing deep injections in the chin area using larger volumes. In these instances, the high cohesivity of VYC‐25L may cause stretching of the mentalis muscle close to the bony insertion; very slow and careful injection allows the soft tissue to expand and the lidocaine in the product to take effect. For any treatment area, overcorrection should be avoided. Indeed, with a structural product like VYC‐25L, it pays to remember the adage that “less is more.”

In addition, aspiration can provide a helpful safety checkpoint in reducing the risk of intravascular placement, although injectors should be wary of complacency—aspiration alone is not sufficient to negate all risk and does not guarantee safe injection [21]. Patients must also be monitored throughout for any signs of vascular injury, particularly unexpected changes in skin color. Moreover, it is important to listen to the patient during and after treatment. Some degree of pain and discomfort may be a normal sequela of filler injection but can also be a first indicator of problems.

Given the firmness and lift capacity of VYC‐25L, it is reasonable to expect a little more post‐procedural swelling than with softer fillers. However, in our experience, this is not a particular concern if the principles outlined above are followed. Moreover, if patients are warned about the possibility of swelling, they are less likely to worry unduly should it occur.

We have heard from some colleagues about concerns around the formation of nonintegrated product nodules with VYC‐25L. However, complete tissue integration has been demonstrated in an ultrasound study [10], and any problems of non‐integration relate primarily to the use of excessively large boluses and overly superficial placement. In our experience, such nodules are rare when key principles of good practice are followed (aseptic technique, appropriate injection plane, slow speed of administration, small product volumes, etc.).

If needed, hyaluronidase can be used to degrade the product, and VYC‐25L is effectively hydrolyzed despite its high HA concentration and tight crosslinking. Indeed, a recent rat study of six HA fillers with similar indications found that all were susceptible to hyaluronidase—including VYC‐25L [24]. There were no significant differences between products in the rate of degradation beyond 1‐h post‐hyaluronidase injection, suggesting that variations in physicochemical properties have no meaningful effect on overall in vivo sensitivity. In our experience, when we need to degrade the product, this is no more difficult with VYC‐25L than with other fillers, particularly when high doses or ultrasound guidance are used. Indeed, two of the present author group sometimes use hyaluronidase to hydrolyze residual product prior to proceeding with VYC‐25L re‐treatment. Nonetheless, new hyaluronidase dosage protocols may be required with structural fillers to ensure that they are rapidly and effectively removed when necessary.

### 2.5 Patient Education

It is rare for new patients to present in the clinic explicitly requesting VYC‐25L (or indeed any other specific filler). This usually only occurs when they have heard about the product from a friend or from online research. However, once they receive VYC‐25L for the first time, it is common for patients to return specifically requesting to have it again.

Irrespective of prior knowledge, it is crucial that appropriate education is provided to patients when using VYC‐25L, particularly with individuals being treated for the first time. For example, they should understand that VYC‐25L is an HA‐based product that mimics a naturally occurring molecule within their body to restore and rebuild the tissue structure, and that in the event of complications, the process can be reversed with hyaluronidase. It is also worthwhile to briefly discuss the physicochemical properties of VYC‐25L that make it well‐suited to volumizing, contouring, and adding definition to the specific facial areas where it will be used.

Duration of effect is another a topic that should be addressed. Patients must appreciate that there is potential for variability between individuals although clinically meaningful benefit often remains for at least 24 months. This also feeds into cost considerations, which are often a key part of the patient discussion. The elongated duration of effect of VYC‐25L—allied to the smaller volumes typically needed compared with other fillers—means that it can provide excellent overall value for money.

It is important to brief patients on the normal sequelae that might be expected during the procedure and in the immediate postinjection period. As noted in the European prospective trial of VYC‐25L, common, but typically minor, localized events may include pain, tenderness, swelling, bruising, edema, and firmness in the treatment area [2, 4]. Patients should be informed that they might experience some pain for a day or two, particularly following treatment of the chin and mandibular line/angle. Individuals who are adequately prepared for these short‐term injection‐site responses are less likely to worry excessively.

In addition, patients must understand that filler injection is a medical procedure, and as with any such procedure, there is always a small risk of more significant complications. In particular, the potential consequences of vascular compromise should be discussed, including necrosis and vision loss. These dangers are not specific to VYC‐25L, and the risk of vision loss is of course lower when treating the jawline or chin as compared with some other areas [25, 26]. Nonetheless, it can be reassuring to discuss with the patient the technical procedures that will be used to minimize vascular risk (e.g., appropriate choice of injection device and plane, slow administration, and aspiration).

Finally, before leaving the clinic, patients should be provided with specific aftercare instructions for optimizing outcomes and minimizing the risk of complications. For example, they may be advised to avoid hot baths/showers, application of make‐up, strenuous exercise, sun exposure, and foods associated with histamine release (such as shellfish) for 1–2 days afterwards. Patients should also refrain from touching or massaging the treated areas. It is advisable that they avoid vaccinations or dental work for 2–4 weeks [21]. Furthermore, patients must be instructed to contact the clinic immediately if they have any concerns about complications, and follow‐up telephone calls should be scheduled in the days following the procedure.

## 3 Conclusions

VYC‐25L is a structural filler designed for facial volumizing and contouring and is our preferred product in appropriate indications (Table 4). It has a unique overall profile based on rheological and physicochemical properties that provide elevated lift capacity and enhanced projection, significant moldability immediately after injection, high levels of tissue integration, reversibility with hyaluronidase, and a long duration of clinical effects (typically at least 24 months). The product is robust and firm but also easy to administer, with a smooth injection profile. By following the key principles outlined here, VYC‐25L can produce natural‐looking and highly durable outcomes without substantial safety concerns.

**TABLE 4:** VYC‐25L: Key takeaways.

VYC‐25L is a unique, robust, structural HA filler with high G′ and high cohesivity—which maximizes lifting capacity and improves resistance to deformation. The rheological properties simulate an injectable implant, and thus chin and jawline augmentation are key indications, although other facial areas can be treated by advanced injectors. Despite its firmness, the VYC‐25L gel is smooth, easy to inject, and moldable immediately postinjection. The product integrates fully into the host tissue within a few weeks. VYC‐25L can be administered using a needle or cannula, and either the subcutaneous or supra‐periosteal plane may be targeted—according to the specific indication, patient goals, injector preference, and safety considerations. The required injection volumes are typically lower with VYC‐25L compared with VYC‐20L. Multi‐session treatment plans may be advisable when using larger amounts. In our experience, VYC‐25L is not more difficult to use and is not associated with increased risk compared with other HA fillers, but adherence to key treatment principles is essential (e.g. aseptic technique, appropriate injection plane, slow speed of administration, small product volumes, etc.). Short‐term, minor, localized sequelae may occur postinjection but can be mitigated with good technique, patient education, and appropriate aftercare. Significant issues with product palpability or non‐integration typically only manifest with excessively large boluses or overly superficially placement—underscoring the importance of good technique. The product can be effectively hydrolyzed by hyaluronidase in the rare instances when this is necessary. In our experience, outcomes with VYC‐25L are natural, durable (lasting at least 24 months), and associated with high levels of patient and physician satisfaction. VYC‐25L is our preferred product in relevant volumizing and contouring indications.

Abbreviations: G′, elastic modulus; HA, hyaluronic acid.

## Author Contributions

All authors contributed to the conception and design of the paper and the interpretations provided; were involved in drafting the manuscript and revising it critically for important intellectual content; gave final approval of the version to be published; and agreed to be accountable for all aspects of the work.

## Ethics Statement

The authors have nothing to report.

## Consent

All of the patients whose photographs are used in this publication provided written informed consent.

## Conflicts of Interest

Dario Bertossi Marshall Murdoch, and Fernando Urdiales‐Gálvez are consultants and speakers for AbbVie. Radina Denkova and David Loh are speakers and trainers for AbbVie. Anna Hoo, Isaac Shturman‐Sirota, and Marcel Vinícius de Aguiar Menezes are speakers for AbbVie. Carola de la Guardia is an employee of Allergan Aesthetics, an AbbVie company.

## Data Availability

The authors have nothing to report.
